# Crystal structure of 4-(naphthalen-2-yl)-2-oxo-6-phenyl-1,2-di­hydro­pyridine-3-carbo­nitrile

**DOI:** 10.1107/S2056989023009180

**Published:** 2023-10-26

**Authors:** Thai Thanh Thu Bui, Dinh Hung Mac, Pham Quang Trung, Chien Thang Pham

**Affiliations:** a Faculty of Chemistry, VNU University of Science, Vietnam National University, Hanoi, 19 Le Thanh Tong, Hanoi, Vietnam; Universidad de Los Andes Mérida, Venezuela

**Keywords:** pyridone, elemental sulfur, three-component one-pot reaction, crystal structure

## Abstract

The synthesis and crystal structure of the title compound, C_22_H_14_N_2_O, are described. The title compound was synthesized by a three-component one-pot reaction in DMSO involving chalcone, cyano­acetamide and elemental sulfur as catalyst. The compound was characterized by spectroscopic methods and single-crystal X-ray diffraction. The structure consists of inversion-related dimers produced by N—H⋯O hydrogen bonding, which further inter­act through π–π contacts.

## Abstract 2

The structure of 4-(naphthalen-2-yl)-2-oxo-6-phenyl-1,2-di­hydro­pyridine-3-carbo­nitrile, prepared by a three-component one-pot reaction, is based on dimers connected by N—H⋯O hydrogen bonds, which also inter­act through π–π contacts.

## 1. Chemical context

Pyridine skeletons play a pivotal role in drug discovery with more than 7000 existing drugs containing this moiety (De *et al.*, 2022 ▸). Recent investigations of 3-cyano­pyrid-2-one derivatives have shown that the unsaturated and cyanide moieties significantly increase their biological activities compared to the original pyridine skeleton. The practical value of these compounds and the broad spectrum of bio­logical activities (ranging from anti­tumor, anti-tuberculosis, anti-inflammatory, anti­microbial activities to anti-SARS-CoV-2) have made the 3-cyano­pyrid-2-ones become the subject of intensive research in pyridine chemistry (Saleh *et al.*, 2021 ▸). Beside their promising biological activity, the 3-cyano­pyrid-2-ones are also used in materials chemistry involving production of OLED devices, dyes, pigments, and other important applications.

Based on a substituted pyridone scaffold, Cheney *et al.* (2007 ▸) identified a novel series of Pim-1 kinase inhibitors that could compete and inter­fere with Pim-1 ATP utilization (Cheney *et al.*, 2007 ▸).

By performing a high throughput screening and an NMR-based fragment screen, 3-cyano­pyridones have been discovered and structurally optimized by hit-to-lead processes to become a novel inhibitor of *M. tuberculosis* thymidylate kinase (Mtb TMK) showing cellular activity against *M. tuberculosis* (Naik *et al.*, 2015 ▸).

Recently, based on the cyclization reaction between 2-nitro-1,3-di­carbonylic compounds and cyano­acetamide, 2-pyridone rings have been synthesized. These compounds are able to inhibit the aggregation of α-synuclein in human cultured cells and prevent the degeneration of dopamine­rgic neurons in the search for novel mol­ecules for the treatment of Parkinson’s disease (Mahía *et al.*, 2021 ▸). The syntheses of 3-cyano­pyrid-2-ones are well documented and highlighted in the review of Litvinov (2006 ▸). These compounds can be synthesized by modification of a substituent in a preformed pyridine substrate or by formation of a C—N bond by a cyclization reaction. During our study on the use of elemental sulfur (Nguyen, 2017*a* ▸,*b* ▸, 2020 ▸) as a versatile sulfurating and oxidizing agent for the syntheses of heterocyclic compounds such as thio­phene, furan, benzo­thia­zine, we noticed that the product of the Michael addition of cyano­acetamide on chalcone can undergo the formation of a C—N bond and aromatization to form the desired 3-cyano­pyrid-2-ones in good yield. 
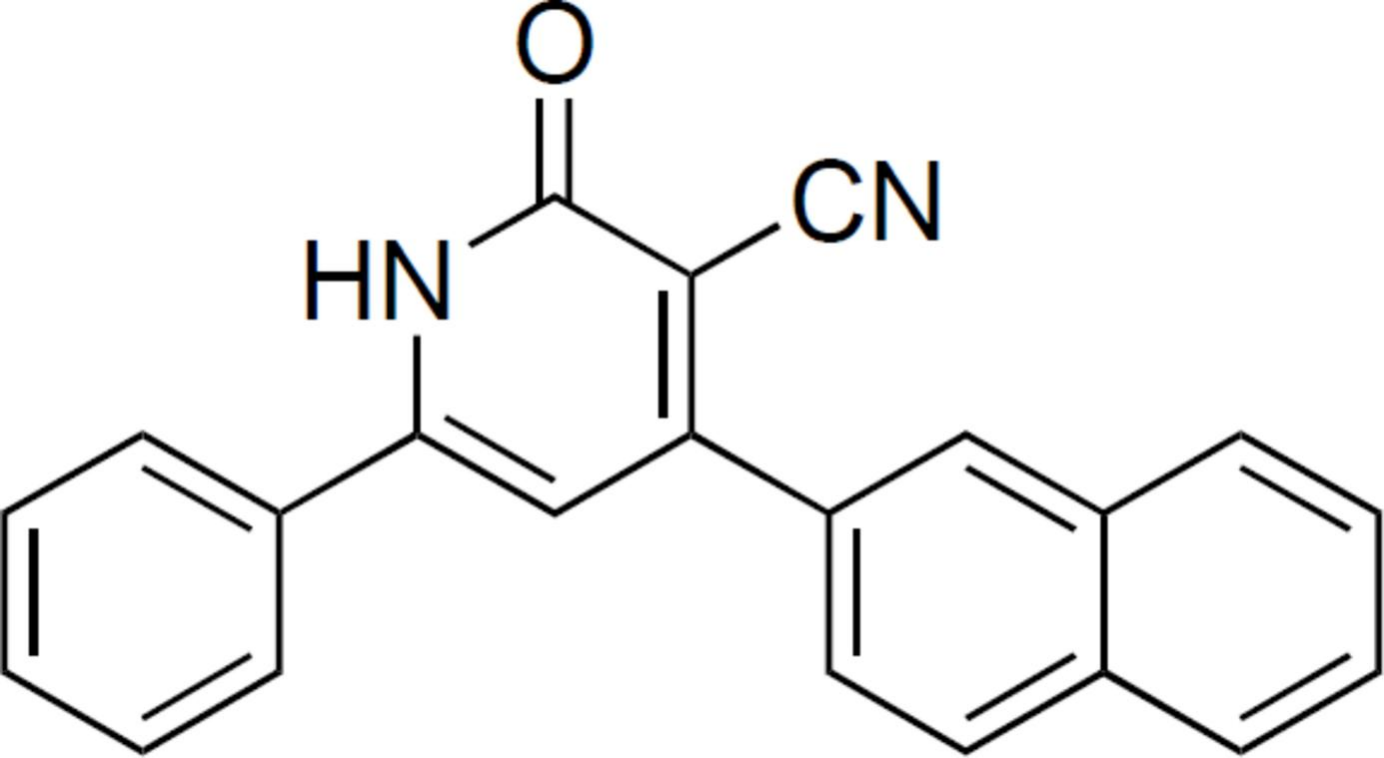


## 2. Structural commentary

**Figure 1:**
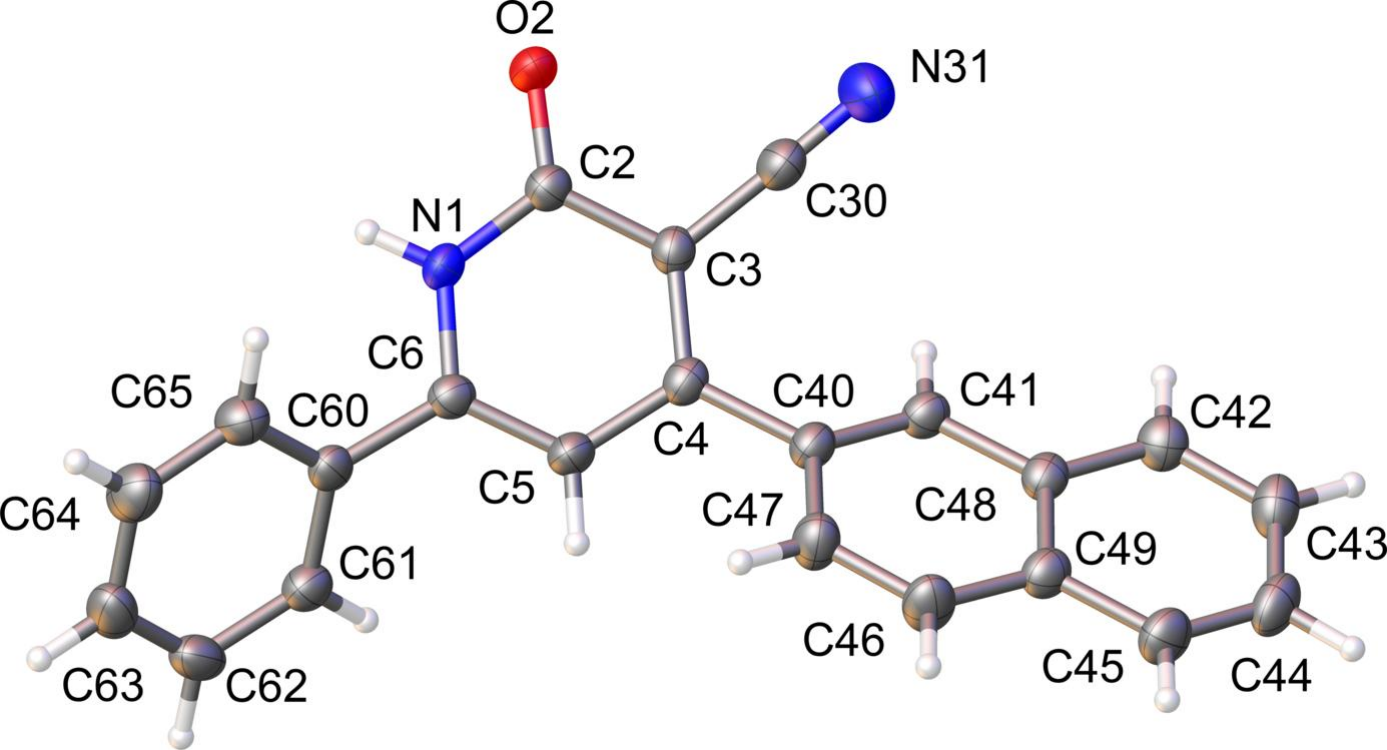
The mol­ecular structure of the title compound, showing the atom labeling. Displacement ellipsoids are drawn at the 50% probability level. Generated with *OLEX2* (Dolomanov *et al.*, 2009 ▸).

The title compound crystallizes in the solvent-free form in the centrosymmetric monoclinic space group *P*2_1_/*n* with one mol­ecule in the asymmetric unit. The mol­ecular structure is shown in Fig. 1 ▸. The *δ*-lactam moiety is almost planar with a maximum deviation from planarity for the N atom of the cyanide group (N31) of 0.047 (2) Å. The phenyl group and the lactam moiety form a dihedral angle of 50.4 (4)° while the naphthyl group is rotated by 35.6 (5)° with respect to the central lactam ring.

## 3. Supra­molecular features

**Figure 2:**
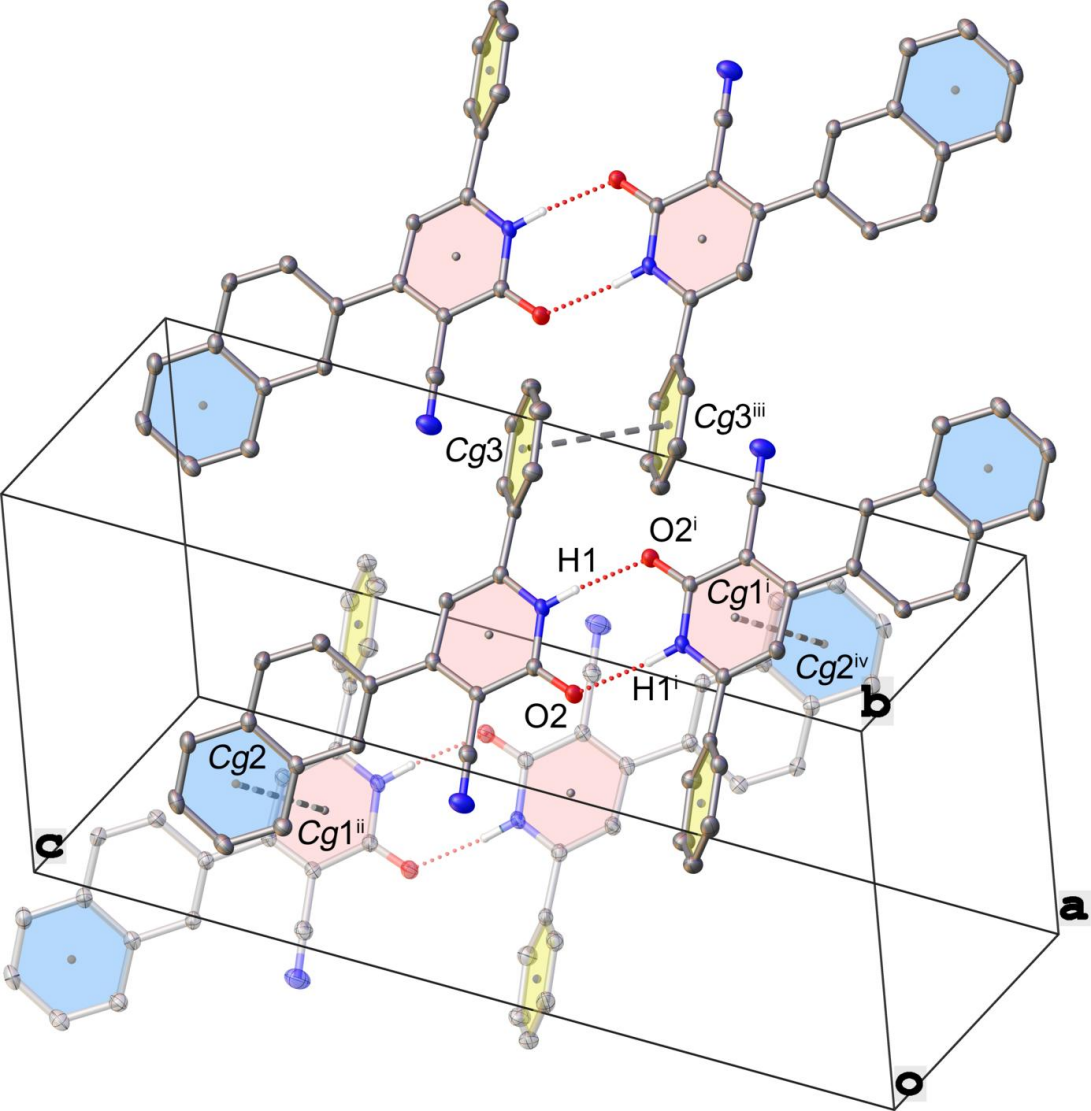
Packing diagram for the title compound, showing N—H⋯O hydrogen bonds and π–π stacking inter­actions. *Cg*1, *Cg*2 and *Cg*3 are the centroids of the lactam N1–C6 ring, the phenyl­ene C42–C49 ring and the phenyl C60–C65 ring, respectively. Generated in *OLEX2* (Dolomanov *et al.*, 2009 ▸). Symmetry codes: (i) −*x* + 2, −*y* + 1, −*z* + 1; (ii) *x* − 1, *y*, *z*; (iii) −*x* + 2, −*y* + 2, −*z* + 1; (iv) −*x* + 1, -*y+*1, −*z* + 1.

In the crystal, mol­ecules form inversion-related dimers *via* N—H⋯O hydrogen bonds (Table 1 ▸ and Fig. 2 ▸). Neighboring dimers inter­act through π–π stacking, namely between the lactam N1–C6 ring and the phenyl­ene C42–C49 ring [centroid-to-centroid distance *Cg*1^ii^⋯*Cg*2 of 3.991 (1) Å and a slippage of 1.968 (3) Å] and between parallel phenyl C60–C65 rings [centroid-to-centroid distance *Cg*3⋯*Cg*3^iii^ of 3.679 (5) Å and a slippage of 1.487 (3) Å].

## 4. Database survey

A search in the Cambridge Structural Database (CSD, Version 5.43, update of November 2022; Groom *et al.*, 2016 ▸) for the 4,6-disubstituted 2-oxo-1,2-di­hydro­pyridine-3-carbo­nitrile subunit reveals eleven hits involving four diaryl derivatives: JINTAC (Rong *et al.*, 2006 ▸, 2007 ▸), PELZIQ, PELZOW and PEQGOL (Chopra *et al.*, 2006 ▸). The rest consists of three compounds containing 4-phenyl-6-alkyl substituents [DOJBUB, DOJCEM (Rai *et al.*, 2014 ▸) and VEXYOP (Rai *et al.*, 2018 ▸)], two compounds possessing 4-alkyl-6-phenyl substituents (DUBXIH; Mishnev *et al.*, 1986 ▸ and RUGVUM; Rai *et al.*, 2015 ▸, 2018 ▸; Chen *et al.*, 2011 ▸) and two dialkyl derivatives (ERISIH; Rybakov *et al.*, 2004 ▸; Elassar, 2011 ▸; Chen *et al.*, 2011 ▸) and GIZBIB (Basheer & Rappoport, 2008 ▸). Across the series of metrics for all structures mentioned, all values regarding the pyridone moiety are in accordance with those reported herein.

## 5. Synthesis and crystallization

A mixture of chalcone (0.2583 g, 1.0 equiv), 2-cyano­acetamide (0.0883 g, 1.05 equiv) and DABCO (0.0224 g, 0.2 equiv) was dissolved in DMSO (0.2 mL). The reaction mixture was heated in a sealed tube at 353 K for 2 h. Then elemental sulfur (0.0064 g, 0.2 equiv) was added to the mixture and the temperature was raised to 393 K for 24 h. After cooling to room temperature, methanol was added to the reaction mixture to precipitate the crude product, which was then filtered and thoroughly washed with methanol and di­chloro­methane. Single crystals suitable for X-ray analysis were obtained by recrystallization of the compound in DMSO/DMF mixture.

^1^H NMR (500 MHz, DMSO-*d*_6_) δ 12.78 (*s*, 1H), 8.35 (*s*, 1H), 8.15–8.00 (*m*, 3H), 7.94 (*d*, *J* = 7.1 Hz, 2H), 7.84 (*dd*, *J* = 8.6, 2.0 Hz, 1H), 7.71–7.47 (*m*, 5H), 6.97 (*s*, 1H).

^13^C NMR (126 MHz, DMSO-*d*_6_) δ 164.2, 162.6, 160.3, 152.0, 133.9, 132.9, 131.7, 130.1, 129.4, 129.2, 129.1, 128.8, 128.8, 128.3, 128.2, 128.1, 127.5, 127.4, 127.1, 125.8, 125.0, 117.1, 107.1.

## 6. Refinement

Crystal data, data collection and structure refinement details are summarized in Table 2 ▸. Positional parameters for the H atom attached to the N atom were refined. All H atoms bonded to C atoms were placed at calculated positions, with C—H = 0.93 Å, and refined as riding with *U*_iso_(H) = 1.2*U*_eq_(C) for C*sp* ^2^—H.

## Supplementary Material

Crystal structure: contains datablock(s) I. DOI: 10.1107/S2056989023009180/dj2070sup1.cif

Structure factors: contains datablock(s) I. DOI: 10.1107/S2056989023009180/dj2070Isup2.hkl

Supporting information file. DOI: 10.1107/S2056989023009180/dj2070Isup3.cdx

Supporting information file. DOI: 10.1107/S2056989023009180/dj2070Isup4.cdx

Supporting information file. DOI: 10.1107/S2056989023009180/dj2070Isup5.cml

CCDC reference: 2302104

Additional supporting information:  crystallographic information; 3D view; checkCIF report

## Figures and Tables

**Table 1:** Hydrogen-bond geometry (Å, °)

*D*—H⋯*A*	*D*—H	H⋯*A*	*D*⋯*A*	*D*—H⋯*A*
N1—H1⋯O2^i^	0.90 (2)	1.91 (2)	2.8096 (18)	172.8 (19)

Symmetry code: (i) 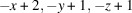 .

**Table 2:** Experimental details

Crystal data	
Chemical formula	C_22_H_14_N_2_O
*M*_r_	322.35
Crystal system, space group	Monoclinic, *P*2_1_/*n*
Temperature (K)	298
*a*, *b*, *c* (Å)	7.0845 (4), 10.4369 (6), 21.6298 (11)
β (°)	91.878 (2)
*V* (Å^3^)	1598.45 (15)
*Z*	4
Radiation type	Mo *K*α
μ (mm^−1^)	0.08
Crystal size (mm)	0.34 × 0.26 × 0.15

Data collection	
Diffractometer	Bruker APEXII CCD
Absorption correction	Multi-scan (*SADABS*; Krause *et al.*, 2015 ▸)
*T*_min_, *T*_max_	0.655, 0.745
No. of measured, independent and observed [*I* > 2σ(*I*)] reflections	18613, 3250, 2531
*R*_int_	0.056
(sin θ/λ)_max_ (Å^−1^)	0.625

Refinement	
*R*[*F* ^2^ > 2σ(*F* ^2^)], *wR*(*F* ^2^), *S*	0.048, 0.123, 1.06
No. of reflections	3250
No. of parameters	230
H-atom treatment	H atoms treated by a mixture of independent and constrained refinement
Δρ_max_, Δρ_min_ (e Å^−3^)	0.18, −0.21

Computer programs: *APEX3* and *SAINT* (Bruker, 2019 ▸), *SHELXT* (Sheldrick, 2015*a* ▸), *SHELXL2018/3* (Sheldrick, 2015*b* ▸), and *OLEX2* (Dolomanov *et al.*, 2009 ▸).

## supplementary crystallographic information

### Crystal data

**Table:**

C_22_H_14_N_2_O	*F*(000) = 672
*M**_r_* = 322.35	*D*_x_ = 1.339 Mg m^−^^3^
Monoclinic, *P*2_1_/*n*	Mo *K*α radiation, λ = 0.71073 Å
*a* = 7.0845 (4) Å	Cell parameters from 7569 reflections
*b* = 10.4369 (6) Å	θ = 2.7–26.3°
*c* = 21.6298 (11) Å	µ = 0.08 mm^−^^1^
β = 91.878 (2)°	*T* = 298 K
*V* = 1598.45 (15) Å^3^	Block, yellow
*Z* = 4	0.34 × 0.26 × 0.15 mm

### Data collection

**Table:**

Bruker APEXII CCD diffractometer	2531 reflections with *I* > 2σ(*I*)
Radiation source: sealed X-ray tube	*R*_int_ = 0.056
φ and ω scans	θ_max_ = 26.4°, θ_min_ = 2.7°
Absorption correction: multi-scan (SADABS; Krause *et al.*, 2015)	*h* = −8→8
*T*_min_ = 0.655, *T*_max_ = 0.745	*k* = −13→13
18613 measured reflections	*l* = −26→26
3250 independent reflections	

### Refinement

**Table:**

Refinement on *F*^2^	Primary atom site location: dual
Least-squares matrix: full	Hydrogen site location: mixed
*R*[*F*^2^ > 2σ(*F*^2^)] = 0.048	H atoms treated by a mixture of independent and constrained refinement
*wR*(*F*^2^) = 0.123	*w* = 1/[σ^2^(*F*_o_^2^) + (0.0459*P*)^2^ + 0.7178*P*] where *P* = (*F*_o_^2^ + 2*F*_c_^2^)/3
*S* = 1.06	(Δ/σ)_max_ < 0.001
3250 reflections	Δρ_max_ = 0.18 e Å^−^^3^
230 parameters	Δρ_min_ = −0.21 e Å^−^^3^
0 restraints	

### Special details

**Table:**

Geometry. All esds (except the esd in the dihedral angle between two l.s. planes) are estimated using the full covariance matrix. The cell esds are taken into account individually in the estimation of esds in distances, angles and torsion angles; correlations between esds in cell parameters are only used when they are defined by crystal symmetry. An approximate (isotropic) treatment of cell esds is used for estimating esds involving l.s. planes.

### Fractional atomic coordinates and isotropic or equivalent isotropic displacement parameters (Å^2^)

**Table:**

	*x*	*y*	*z*	*U*_iso_*/*U*_eq_	
O2	0.82728 (17)	0.39070 (12)	0.51570 (6)	0.0445 (3)	
N1	0.8282 (2)	0.59995 (14)	0.54490 (6)	0.0335 (3)	
C6	0.7537 (2)	0.70213 (16)	0.57481 (8)	0.0329 (4)	
C2	0.7497 (2)	0.47978 (16)	0.54354 (8)	0.0331 (4)	
C48	0.0535 (2)	0.45419 (16)	0.68796 (8)	0.0334 (4)	
C60	0.8580 (2)	0.82447 (16)	0.57434 (8)	0.0341 (4)	
C4	0.5045 (2)	0.56580 (17)	0.61101 (8)	0.0327 (4)	
C3	0.5797 (2)	0.46509 (16)	0.57786 (8)	0.0322 (4)	
C41	0.1982 (2)	0.46820 (17)	0.64419 (8)	0.0342 (4)	
H41	0.191623	0.420700	0.607804	0.041*	
C40	0.3471 (2)	0.55046 (16)	0.65470 (8)	0.0340 (4)	
C5	0.5920 (2)	0.68616 (17)	0.60673 (8)	0.0366 (4)	
H5	0.538843	0.756446	0.626038	0.044*	
C61	0.7640 (3)	0.93869 (17)	0.56255 (8)	0.0409 (4)	
H61	0.635429	0.937948	0.552319	0.049*	
C49	0.0619 (2)	0.52910 (18)	0.74242 (8)	0.0381 (4)	
N31	0.4577 (3)	0.23249 (18)	0.58144 (10)	0.0640 (5)	
C42	−0.0972 (2)	0.36732 (18)	0.67854 (9)	0.0411 (4)	
H42	−0.101608	0.315499	0.643546	0.049*	
C65	1.0507 (3)	0.82723 (19)	0.58974 (9)	0.0443 (5)	
H65	1.116142	0.751180	0.597029	0.053*	
C47	0.3542 (3)	0.62309 (19)	0.71018 (9)	0.0437 (5)	
H47	0.455112	0.678437	0.717990	0.052*	
C45	−0.0852 (3)	0.51704 (19)	0.78517 (9)	0.0459 (5)	
H45	−0.081273	0.565435	0.821287	0.055*	
C62	0.8604 (3)	1.05386 (18)	0.56591 (10)	0.0499 (5)	
H62	0.797354	1.130175	0.557074	0.060*	
C30	0.5102 (2)	0.33702 (19)	0.58021 (9)	0.0422 (5)	
C43	−0.2373 (3)	0.3587 (2)	0.72060 (9)	0.0478 (5)	
H43	−0.336678	0.301691	0.713812	0.057*	
C46	0.2157 (3)	0.61315 (19)	0.75229 (9)	0.0467 (5)	
H46	0.222902	0.662601	0.788055	0.056*	
C44	−0.2314 (3)	0.4353 (2)	0.77365 (9)	0.0490 (5)	
H44	−0.328752	0.430277	0.801378	0.059*	
C63	1.0496 (3)	1.0555 (2)	0.58234 (10)	0.0541 (6)	
H63	1.113658	1.133087	0.585465	0.065*	
C64	1.1440 (3)	0.9430 (2)	0.59413 (11)	0.0545 (5)	
H64	1.271945	0.944829	0.605168	0.065*	
H1	0.937 (3)	0.610 (2)	0.5249 (10)	0.053 (6)*	

### Atomic displacement parameters (Å^2^)

**Table:**

	*U*^11^	*U*^22^	*U*^33^	*U*^12^	*U*^13^	*U*^23^
O2	0.0441 (7)	0.0351 (7)	0.0557 (8)	−0.0034 (6)	0.0221 (6)	−0.0099 (6)
N1	0.0315 (7)	0.0344 (8)	0.0352 (8)	−0.0007 (6)	0.0113 (6)	−0.0002 (6)
C6	0.0349 (9)	0.0320 (9)	0.0320 (9)	0.0030 (7)	0.0059 (7)	0.0001 (7)
C2	0.0339 (8)	0.0335 (9)	0.0321 (9)	0.0007 (7)	0.0062 (7)	−0.0006 (7)
C48	0.0313 (8)	0.0351 (9)	0.0341 (9)	0.0028 (7)	0.0032 (7)	0.0049 (7)
C60	0.0378 (9)	0.0335 (9)	0.0316 (9)	−0.0009 (7)	0.0124 (7)	−0.0017 (7)
C4	0.0289 (8)	0.0372 (9)	0.0322 (9)	0.0016 (7)	0.0049 (7)	0.0018 (7)
C3	0.0306 (8)	0.0337 (9)	0.0326 (9)	−0.0018 (7)	0.0045 (7)	0.0019 (7)
C41	0.0349 (9)	0.0365 (9)	0.0315 (9)	0.0020 (7)	0.0064 (7)	−0.0016 (7)
C40	0.0317 (8)	0.0353 (9)	0.0355 (9)	0.0019 (7)	0.0076 (7)	0.0014 (7)
C5	0.0366 (9)	0.0322 (9)	0.0417 (10)	0.0027 (7)	0.0130 (7)	−0.0022 (8)
C61	0.0408 (10)	0.0387 (10)	0.0441 (10)	0.0038 (8)	0.0126 (8)	0.0003 (8)
C49	0.0398 (9)	0.0406 (10)	0.0345 (9)	0.0030 (8)	0.0085 (7)	0.0028 (8)
N31	0.0523 (11)	0.0456 (11)	0.0951 (15)	−0.0076 (9)	0.0158 (10)	−0.0058 (10)
C42	0.0389 (9)	0.0437 (11)	0.0406 (10)	−0.0026 (8)	0.0018 (8)	0.0007 (8)
C65	0.0406 (10)	0.0400 (11)	0.0526 (12)	0.0025 (8)	0.0060 (8)	−0.0039 (9)
C47	0.0408 (10)	0.0473 (11)	0.0436 (11)	−0.0099 (9)	0.0091 (8)	−0.0063 (9)
C45	0.0489 (11)	0.0520 (12)	0.0377 (10)	0.0004 (9)	0.0142 (8)	0.0008 (9)
C62	0.0655 (13)	0.0310 (10)	0.0546 (12)	0.0045 (9)	0.0220 (10)	−0.0019 (9)
C30	0.0347 (9)	0.0432 (11)	0.0494 (11)	−0.0003 (8)	0.0102 (8)	−0.0032 (9)
C43	0.0388 (10)	0.0557 (12)	0.0494 (11)	−0.0077 (9)	0.0057 (8)	0.0098 (10)
C46	0.0526 (11)	0.0510 (12)	0.0373 (10)	−0.0099 (9)	0.0130 (8)	−0.0124 (9)
C44	0.0411 (10)	0.0631 (13)	0.0438 (11)	0.0025 (10)	0.0166 (8)	0.0126 (10)
C63	0.0601 (13)	0.0418 (12)	0.0618 (13)	−0.0143 (10)	0.0225 (10)	−0.0150 (10)
C64	0.0412 (11)	0.0548 (13)	0.0680 (14)	−0.0081 (10)	0.0082 (10)	−0.0142 (11)

### Geometric parameters (Å, º)

**Table:**

O2—C2	1.245 (2)	C61—C62	1.383 (3)
N1—C6	1.363 (2)	C49—C45	1.421 (2)
N1—C2	1.372 (2)	C49—C46	1.410 (3)
N1—H1	0.90 (2)	N31—C30	1.153 (3)
C6—C60	1.475 (2)	C42—H42	0.9300
C6—C5	1.367 (2)	C42—C43	1.371 (3)
C2—C3	1.444 (2)	C65—H65	0.9300
C48—C41	1.425 (2)	C65—C64	1.379 (3)
C48—C49	1.413 (3)	C47—H47	0.9300
C48—C42	1.411 (2)	C47—C46	1.364 (2)
C60—C61	1.385 (2)	C45—H45	0.9300
C60—C65	1.395 (3)	C45—C44	1.359 (3)
C4—C3	1.388 (2)	C62—H62	0.9300
C4—C40	1.494 (2)	C62—C63	1.375 (3)
C4—C5	1.405 (2)	C43—H43	0.9300
C3—C30	1.426 (3)	C43—C44	1.398 (3)
C41—H41	0.9300	C46—H46	0.9300
C41—C40	1.373 (2)	C44—H44	0.9300
C40—C47	1.419 (3)	C63—H63	0.9300
C5—H5	0.9300	C63—C64	1.370 (3)
C61—H61	0.9300	C64—H64	0.9300
			
C6—N1—C2	124.23 (14)	C46—C49—C48	118.80 (15)
C6—N1—H1	119.1 (13)	C46—C49—C45	122.42 (17)
C2—N1—H1	116.7 (13)	C48—C42—H42	119.8
N1—C6—C60	118.17 (14)	C43—C42—C48	120.48 (18)
N1—C6—C5	119.25 (16)	C43—C42—H42	119.8
C5—C6—C60	122.49 (15)	C60—C65—H65	120.1
O2—C2—N1	120.53 (15)	C64—C65—C60	119.83 (18)
O2—C2—C3	123.93 (16)	C64—C65—H65	120.1
N1—C2—C3	115.50 (15)	C40—C47—H47	119.4
C49—C48—C41	119.03 (16)	C46—C47—C40	121.16 (17)
C42—C48—C41	121.83 (16)	C46—C47—H47	119.4
C42—C48—C49	119.14 (15)	C49—C45—H45	119.8
C61—C60—C6	120.54 (16)	C44—C45—C49	120.49 (18)
C61—C60—C65	119.16 (17)	C44—C45—H45	119.8
C65—C60—C6	120.18 (16)	C61—C62—H62	120.0
C3—C4—C40	123.54 (15)	C63—C62—C61	119.97 (19)
C3—C4—C5	117.71 (14)	C63—C62—H62	120.0
C5—C4—C40	118.54 (15)	N31—C30—C3	178.4 (2)
C4—C3—C2	121.73 (15)	C42—C43—H43	119.8
C4—C3—C30	123.54 (15)	C42—C43—C44	120.30 (18)
C30—C3—C2	114.38 (15)	C44—C43—H43	119.8
C48—C41—H41	119.4	C49—C46—H46	119.5
C40—C41—C48	121.29 (16)	C47—C46—C49	121.03 (17)
C40—C41—H41	119.4	C47—C46—H46	119.5
C41—C40—C4	123.20 (15)	C45—C44—C43	120.75 (17)
C41—C40—C47	118.67 (15)	C45—C44—H44	119.6
C47—C40—C4	118.11 (15)	C43—C44—H44	119.6
C6—C5—C4	121.42 (16)	C62—C63—H63	119.9
C6—C5—H5	119.3	C64—C63—C62	120.21 (19)
C4—C5—H5	119.3	C64—C63—H63	119.9
C60—C61—H61	119.9	C65—C64—H64	119.7
C62—C61—C60	120.29 (18)	C63—C64—C65	120.52 (19)
C62—C61—H61	119.9	C63—C64—H64	119.7
C48—C49—C45	118.78 (17)		
			
O2—C2—C3—C4	176.33 (17)	C41—C48—C49—C46	−1.4 (3)
O2—C2—C3—C30	3.0 (3)	C41—C48—C42—C43	−178.05 (17)
N1—C6—C60—C61	135.08 (17)	C41—C40—C47—C46	−0.7 (3)
N1—C6—C60—C65	−49.1 (2)	C40—C4—C3—C2	−170.35 (16)
N1—C6—C5—C4	2.1 (3)	C40—C4—C3—C30	2.4 (3)
N1—C2—C3—C4	−1.4 (2)	C40—C4—C5—C6	170.28 (16)
N1—C2—C3—C30	−174.79 (15)	C40—C47—C46—C49	0.8 (3)
C6—N1—C2—O2	−179.26 (16)	C5—C6—C60—C61	−48.6 (2)
C6—N1—C2—C3	−1.4 (2)	C5—C6—C60—C65	127.28 (19)
C6—C60—C61—C62	176.01 (17)	C5—C4—C3—C2	4.3 (2)
C6—C60—C65—C64	−174.71 (18)	C5—C4—C3—C30	177.09 (17)
C2—N1—C6—C60	177.59 (15)	C5—C4—C40—C41	148.18 (17)
C2—N1—C6—C5	1.1 (3)	C5—C4—C40—C47	−32.9 (2)
C48—C41—C40—C4	178.35 (16)	C61—C60—C65—C64	1.2 (3)
C48—C41—C40—C47	−0.5 (3)	C61—C62—C63—C64	1.4 (3)
C48—C49—C45—C44	−0.3 (3)	C49—C48—C41—C40	1.6 (3)
C48—C49—C46—C47	0.3 (3)	C49—C48—C42—C43	2.2 (3)
C48—C42—C43—C44	−0.6 (3)	C49—C45—C44—C43	2.0 (3)
C60—C6—C5—C4	−174.25 (16)	C42—C48—C41—C40	−178.13 (16)
C60—C61—C62—C63	−1.4 (3)	C42—C48—C49—C45	−1.8 (3)
C60—C65—C64—C63	−1.2 (3)	C42—C48—C49—C46	178.30 (17)
C4—C40—C47—C46	−179.62 (18)	C42—C43—C44—C45	−1.6 (3)
C3—C4—C40—C41	−37.2 (3)	C65—C60—C61—C62	0.1 (3)
C3—C4—C40—C47	141.73 (18)	C45—C49—C46—C47	−179.63 (19)
C3—C4—C5—C6	−4.7 (3)	C62—C63—C64—C65	−0.1 (3)
C41—C48—C49—C45	178.47 (16)	C46—C49—C45—C44	179.63 (19)

### Hydrogen-bond geometry (Å, º)

**Table:**

*D*—H···*A*	*D*—H	H···*A*	*D*···*A*	*D*—H···*A*
N1—H1···O2^i^	0.90 (2)	1.91 (2)	2.8096 (18)	172.8 (19)

Symmetry code: (i) −*x*+2, −*y*+1, −*z*+1.
